# Effect of the Manufacturing Process on the Microbiota, Organoleptic Properties and Volatilome of Three Salmon-Based Products

**DOI:** 10.3390/foods10112517

**Published:** 2021-10-20

**Authors:** Norman Wiernasz, Frédérique Gigout, Mireille Cardinal, Josiane Cornet, Jens Rohloff, Philippe Courcoux, Evelyne Vigneau, Sigurlaug Skírnisdottír, Delphine Passerini, Marie-France Pilet, Françoise Leroi

**Affiliations:** 1 IFREMER, BRM, EM3B Laboratory, F-44300 Nantes, France; norman.wiernasz@biofortis.fr (N.W.); frederique.gigout@ifremer.fr (F.G.); mireille.cardinal@ifremer.fr (M.C.); josiane.cornet1@sfr.fr (J.C.); delphine.passerini@ifremer.fr (D.P.); 2 UMR 1014, Secalim, INRAE, Oniris, 44307 Nantes, France; marie-france.pilet@inrae.fr; 3 NTNU, Department of Biology, 7491 Trondheim, Norway; jens.rohloff@ntnu.no; 4 StatSC, Oniris, INRAE, 44322 Nantes, France; philippe.courcoux@oniris-nantes.fr (P.C.); evelyne.vigneau@oniris-nantes.fr (E.V.); 5 Matıs, Research and Innovation, Exploitation and Utilization of Genetic Resources, 101-155 Reykjavik, Iceland; sigurlaug@matis.is

**Keywords:** cold-smoked salmon, gravlax, seafood, microbiology, metabarcoding, 16S rRNA gene, volatile organic compound, sensory analysis, quality

## Abstract

Lightly preserved seafood products, such as cold-smoked fish and fish gravlax, are traditionally consumed in Europe and are of considerable economic importance. This work aimed to compare three products that were obtained from the same batch of fish: cold-smoked salmon (CSS) stored under vacuum packaging (VP) or a modified atmosphere packaging (MAP) and VP salmon dill gravlax (SG). Classical microbiological analyses and 16S rRNA metabarcoding, biochemical analyses (trimethylamine, total volatile basic nitrogen (TVBN), biogenic amines, pH, volatile organic compounds (VOCs)) and sensory analyses (quantitative descriptive analysis) were performed on each product throughout their storage at a chilled temperature. The three products shared the same initial microbiota, which were mainly dominated by *Photobacterium*, *Lactococcus* and *Lactobacillus* genera. On day 28, the VP CSS ecosystem was mainly composed of *Photobacterium* and, to a lesser extent, *Lactococcus* and *Lactobacillus* genera, while *Lactobacillus* was dominant in the MAP CSS. The diversity was higher in the SG, which was mainly dominated by *Enterobacteriaceae*, *Photobacterium, Lactobacillus* and *Lactococcus*. Although the sensory spoilage was generally weak, gravlax was the most perishable product (slight increase in amine and acidic off-odors and flavors, fatty appearance, slight discoloration and drop in firmness), followed by the VP CSS, while the MAP CSS did not spoil. Spoilage was associated with an increase in the TVBN, biogenic amines and spoilage associated VOCs, such as decanal, nonanal, hexadecanal, benzaldehyde, benzeneacetaldehyde, ethanol, 3-methyl-1-butanol, 2,3-butanediol, 1-octen-3-ol, 2-butanone and 1-octen-3-one. This study showed that the processing and packaging conditions both had an effect on the microbial composition and the quality of the final product.

## 1. Introduction

Over the last decade, high-added-value seafood products, including smoked fish, gravlax and sea salads, have been gaining popularity in Europe and have dedicated refrigerated shelves in supermarkets. Cold-smoked salmon (CSS) is one of the best-selling products. The European production was estimated at 175,000 tons in 2019 for a trade value of 2.77 billion EUR, where the domestic demand mainly included France, the United Kingdom, Spain and Poland [1,2].

The smoking process is one of the oldest methods used to preserve fish. The process consists of three steps, namely, salting (dry salt or brine), drying and smoking sensu stricto. The smoking temperature varies from 22–25 °C for CSS to 70 °C for hot-smoked salmon. The production of gravlax includes just the stages of salting, with a mixture of dried salt, sugar, spices and herbs, and drying. CSS and salmon gravlax (SG) are lightly processed products (NaCl < 6% in the water phase, pH > 5, phenolic compounds < 20 mg/kg for CSS) that are usually stored at refrigerated temperatures under vacuum packaging (VP). Although not a common practice, a modified atmosphere (MAP) is sometimes used to store such products.

The light processing step does not eliminate microorganisms. Some of them can grow during storage, leading to the production of off-odors and flavors and a pasty texture. The sensory acceptability usually ranges between 2 to 4 weeks for gravlax and up to 6 weeks for CSS. It is generally admitted that the CSS initial microbiota is dominated by Gram-negative bacteria that are typically associated with fresh raw fish, such as *Photobacterium*, *Shewanella*, *Vibrio* and *Yersinia*. During storage, Gram-positive bacteria, especially lactic acid bacteria (LAB), such as *Carnobacterium* and *Lactobacillus*, become predominant with concentrations around 10^7^−10^9^ CFU/g. However, Enterobacteriaceae, *Photobacterium* and *Brochothrix* can also be present at sufficient levels to induce spoilage [3,4,5,6,7,8,9,10,11,12,13]. Although rarely quantified, yeasts are found throughout storage as subdominant microbiota (10^4^ CFU/g) [3]. In SG and trout gravlax, Enterobacteriaceae dominate the ecosystem but LAB and to a lesser extent *Brochothrix* are also present [14,15,16].

Recently, new culture-independent methods, such as 16S rRNA gene Illumina metabarcoding or 4-5-4 pyrosequencing, were used to obtain a full and clear picture of the microbial ecosystem composition and dynamics in foods. To date, only three studies considered VP CSS and involved twenty-two batches from different smokehouses and salmon origins analyzed throughout the storage [17,18,19]. A core microbiota between samples could clearly be found, even if some variation was observed. The initial microbiota was generally dominated by *Photobacterium phosphoreum*/*kishitanii*, *Aliivibrio* sp., *Lactobacillus sakei*, *Brochothrix thermosphacta* and, to a lesser extent, a few LAB, such as *Leuconostoc gasicomitatum*, *Lactococcus piscium*, *Carnobacterium divergens* and *Carnobacterium maltaromaticum*. The storage conditions exerted strong selective pressure on the initial microbiota and, at the spoiling date, the composition was highly variable between samples. Some batches were dominated by *Staphylococcus equorum* and LAB (*Lactobacillus curvatus*, *Lactococcus lactis*, *C. divergens*, *C. maltaromaticum*), while others were dominated by *B. thermosphacta* and LAB (*C. maltaromaticum*, *Lactobacillus fuchuensis*) or by *P. phosphoreum*/*kishitanii* alone. Enterobacteriaceae (*Serratia proteamaculans* and *Hafnia alvei*) were also detected in some samples, as well as *Psychrobacter*, *Shewanella*, *Salinivibrio* and unexpected *Pantoea* genera. No study was found on MAP CSS dices, and only one concerned a single batch of SG [16]. Its initial ecosystem was mainly composed of *Photobacterium* and *Pseudomonas* and many subdominant operational taxonomic units (OTU). During VP storage, the number of OTU decreased and *Photobacterium*, *Serratia*/*Yersinia* and *Vibrio* dominated the ecosystem, as well as *Lactococcus*, *Carnobacterium*, *Lactobacillus* and *Aerococcus*.

The objective of this study was to gain deeper knowledge of the quality of three salmon-based products, namely, salmon gravlax packed under VP (SG), CSS packed under VP (VP CSS) and CSS packed under MAP (MAP CSS), using classical and omics tool analysis. A polyphasic approach that included measures of the microbial ecosystem (cultural and metabarcoding), organoleptic quality, biogenic amines, total volatile basic nitrogen (TVBN), pH and volatilome was used. A new statistical approach (ComDim) was tested to draw links between the heterogeneous data to better understand the spoilage. The impact of different manufacturing processes or storage conditions was also evaluated, as the three products were obtained from the same batch of raw salmon.

## 2. Material and Methods

### 2.1. Salmon Products, Raw Material Process and Storage

Raw salmon fish (*Salmo salar*) were purchased in Norway and processed on the same industrial site in France. Whole gutted salmon were filleted and stored at 4 °C in France, 4 days after slaughter. Salmon dill gravlax (SG) and smoked salmon were all processed the next day.

Salmon gravlax: Fillets were cured with a mix of salt, sugar, black pepper and dill (internal recipe) over 14 h at 6 °C. They were then rinsed, sliced and packed in blisters under vacuum as 120 g portions of 8 slices.

Smoked salmon: Fillets were dry salted over 10 h at 4 °C. They were then rinsed and dried for 2.5 h at 23 °C. Fillets were cold smoked over 6 h at 23 °C and left in a maturation chamber for 2 days at temperatures of 0–4 °C. Matured fillets were sliced and vacuum packed as 150 g portions of 5 slices for smoked salmon under vacuum (VP CSS). For smoked salmon under a modified atmosphere (MAP CSS), the fillets were cut into cubes and packed under a modified atmosphere (40% CO_2_/60% N_2_) as a 500 g portion.

Immediately after packaging, the products were transported to the laboratory under refrigerated conditions.

### 2.2. Sampling Dates and Type of Analyses

The commercial best before date for MAP CSS and SG was 28 days and 35 days for VP CSS. However, to push spoilage through extended storage, the MAP CSS and SG were stored for 35 days and the VP CSS for 49 days. All products were incubated at 4 °C for 1 week and then at 8 °C for the rest of the storage period. From T0, which corresponded to the first day of the experiment, and every 7 days, samples were withdrawn for microbial enumeration, biochemical analyses (trimethylamine (TMA), TVBN, biogenic amines, pH, volatile organic compounds (VOCs) composition) and sensory analyses. Ecosystem monitoring through a metabarcoding approach was performed for the 3 products at T0 and after 14 and 28 days of storage. Except for the sensory assessment (see Section 2.5), all analyses were performed in triplicates.

### 2.3. Classical Microbiological Analysis

At each sampling date, 20 g of product were aseptically stomached (Stomacher 400 circulator, Seward Medical, London, UK) for 2 min with 80 mL of refrigerated sterile tryptone salt (TS) solution (Biokar Diagnostics, Beauvais, France) with 1% Tween 80 (Grosseron, Saint-Herblain, France). After 30 min of revivification at room temperature, appropriate dilutions made in TS-Tween were poured or spread plated for enumeration of the total viable count (TVC) and total LAB, *B. thermosphacta* and Enterobacteriaceae counts, according to the conditions described by Wiernasz et al. [16].

### 2.4. Metabarcoding of 16S rRNA Gene

Total bacterial DNA extraction and 16S rRNA sequencing: Bacterial DNA was extracted from the stomached solution according to the modified and optimized protocol with a MasterPure™ Gram Positive DNA purification kit (Epicentre, Illumina, Madison, WI, USA), as described by Wiernasz et al. [16]. The EMP primers set (515f/806r) from [20] was used to amplify the hypervariable V4 region of the bacterial 16S rRNA gene using polymerase chain reaction (PCR). Amplification and data treatment were performed as described by Wiernasz et al. [16].

Bioinformatics processing of the data: The bioinformatic processing of the data was done as described by Wiernasz et al. [16]. Briefly, FastQC [21] was applied on demultiplexed reads (around 300 pb) to check their quality. Reads R1 were trimmed after 280 pb and reads R2 after 230 pb with FASTX-trimmer from the FASTX-Toolkit [22]. Reads were then processed with the FROGS pipeline [23] using Flash [24] with 10% mismatches, Cutadapt [25] and clustering with Swarm [26] according to Escudié et al. [23] recommendations. Clusters were then filtered regarding their abundance and occurrence by representing a minimum of 0.005% of all sequences and being present in at least 3 samples. Clusters affiliation was performed with blastn+ [27] against 16S Silva database version 123 [28], and the OTU were filtered depending on the identity and coverage value of 100%.

Downstream analyses were performed on rarified counts with R version 3.4.4 [29] under the RStudio environment version 1.1.442 [30]. For the metabarcoding data, alpha and beta diversity analyses were performed using the R packages Phyloseq [31] and vegan version 2.5.7 [32]. For the beta diversity, a multidimensional scaling (MDS) analysis was conducted based on the samples’ dissimilarity matrix, which was calculated using the Bray–Curtis distance. The R package DESeq2 [33] was used to perform the differential abundance analysis on samples raw counts that were normalized following an rlog transformation (log_2_(x + 1)). All graphical visualizations were performed with the R package ggplot2 [34].

Fastq file availability: The raw fastq files were deposited on Ifremer’s Sextant database and are accessible through the following DOI number: https://doi.org/10.12770/1edc84cf-f3ef-47be-a096-e820b3806aec (accessed on 16 October 2018).

### 2.5. Sensory Analysis

At each sampling date, portions of the SG, VP CSS and MAP CSS were collected and kept frozen at −80 °C until analysis. For each product, a conventional sensory profiling test was conducted according to ISO 13299 [35]. The sensory evaluation was performed using an internal trained panel of 17 judges that were experienced in seafood, especially in salmon products [10,36]. During the sessions, the panelists were asked to assess the global spoilage based on aspect and off-odors perception. Then, the products were described according to a list of relevant sensory descriptors for odor (fatty fish, acid, amine, smoke and dill), appearance (fatty and orange color), texture (greasy film, firm, melting and pasty texture) and flavor (acid, salty, amine, fish, smoked, sweet and dill). Both the global spoilage and descriptors were scored depending on their intensity on a continuous scale from 0 (low intensity) up to 10 (high intensity). A product was considered strongly spoiled and unfit for consumption when the global spoilage level exceeded a threshold value of 6.

The day before the sensory evaluation, samples were thawed overnight at 4 °C. Sessions were performed in individual partitioned booths, as described in the procedure NF V-09-105 [37] and equipped with a computerized system (Fizz, Biosystèmes, Couternon, France). Each panelist received one slice of the SG and 15 g of MAP CSS dices, where both were presented in a covered plastic container. For the VP CSS, a half slice (around 15 g) was repacked and presented in aluminum foil. Samples were assigned with 3-digit numbers and randomized regarding the order of presentation for each panelist. For each matrix, odor and appearance were assessed for all sampling dates, while flavor and texture were assessed on 3 dates: days 1, 21 and 28 for the SG; days 1, 21 and 35 for the MAP CSS; and days 1, 28 and 35 for the VP CSS. 

Normalized principal component analyses (PCAs) were performed on the sensory descriptors’ mean scores using the R package ggfortify [38]. In addition, a two-way analysis of variance (ANOVA) was applied to the panelists’ descriptor scores using products (with time) and panelists as independent factors. Significant differences between means were determined using Duncan’s multiple range test (*p*-value < 0.05) (Fizz software, Biosystèmes, Couternon, France).

### 2.6. Biochemical Analysis

#### 2.6.1. Physicochemical Parameters

The TVBN and TMA were quantified at each sampling date from 80 g of minced product according to the Conway microdiffusion method [39]. The pH value was measured directly after the microbiological analysis in the stomached solution with a pH meter (Mettler Toledo AG, Schwerzenbach, Switzerland).

#### 2.6.2. Biogenic Amines Measurement

Ten milliliters of the stomached solution (see Section 2.3) was mixed with 5 mL of a trichloroacetic acid solution at 12% (Panreac, Darmstadt, Germany). The samples were kept frozen at −20 °C until analysis. Eight biogenic amines (tryptamine, 2-phenylethylamine, putrescine, cadaverine, histamine, tyramine, spermidine, spermine) were quantified using high-pressure liquid chromatography (HPLC) following Wiernasz et al.’s [40] methodology.

#### 2.6.3. Headspace–Solid-Phase Microextraction (HS-SPME) and Gas Chromatography/Mass Spectrometry (GC/MS) Analysis of the Volatilome

For each sampling date for the 3 products, portions of 20 g were withdrawn and stocked under VP at −40 °C. Eight salmon flesh cylinders were sampled across the frozen product using a pre-cooled metal cork borer and immediately pooled to make up 1 g of an analysis sample. Samples were kept frozen in 4 mL vials with a screw cap and PTFE/silicone septum at −40 °C prior to extraction and analysis. For each sample (time point and treatment), 3 independent analysis samples (triplicate) were prepared. Prior to the volatile extraction, a 30% *w*/*v* NaCl solution (H_2_O) was added to the sample, which was finally minced using a high-speed homogenizer. HS-SPME was applied for the extraction of VOCs using a manual SPME holder with a PDMS/DVB-coated 65 μm fiber (Supelco Inc., Bellefonte, PA, USA). Prior to the extraction, the SPME fiber was conditioned in the injection port of the GC according to the instructions provided by the supplier. The SPME fiber was exposed to the atmosphere in the closed sample vial for 30 min while keeping the vial in an isothermal condition at 50 °C in a water bath. Samples were constantly agitated using a magnetic stirrer during extraction. 

An Agilent 6890/5975 GC/MS (Agilent Technologies Inc., Palo Alto, CA, USA) was used for all analyses. Analytes absorbed on the SPME fiber were desorbed in the injection port for 3 min under splitless conditions. GC separations were carried out using an apolar HP-5MS capillary column (30 m × 0.25 mm and film thickness 0.25 µm). The injection temperature was 220 °C and the interface was set to 220 °C. The carrier gas was He at a constant flow rate of 1 mL/min. The GC temperature was ramped from 40 to 211 °C at a rate of 4.5 °C/min, then raised at a rate of 50 °C/min and finally held at 220 °C (total run time: 40 min). The MS source was adjusted to 230 °C and a mass range of *m*/*z* 35–350 was recorded. Mass spectra were acquired in electron impact ionization (EI) mode at 70 eV.

GC/MS chromatograms were visualized using the following GC/MS software packages: Agilent ChemStation software (Agilent Technologies, Waldbronn, Germany), AMDIS software (version 2.71; National Institute of Standards and Technology, Boulder, CO, USA) and the open-source program OpenChrom Community Edition Alder (version 1.2.0) (Lablicate GmbH, Hamburg, Germany; https://lablicate.com/platform/openchrom)(accessed on 10 August 2021). 

Tentative identification of compounds was carried out using (a) MS libraries, such as NIST05 spectral library (National Institute of Standards and Technology, Gaithersburgh, MD), the NIST Chemistry WebBook (https://webbook.nist.gov/chemistry)(accessed on 10 August 2021) and a customized in-house MS library of VOCs; in combination with (b) linear retention indices (LRI), based on a homologous series of even-numbered n-alkanes (C8 to C24); and (c) LRIs found in the literature and the NIST Chemistry WebBook. GC/MS data integration, normalization (total signal) and alignment were carried out using the Metalign software (RIKILT WUR, Wageningen, The Netherlands). The detected analytes concentrations were quantitatively estimated based on an internal standard (BHT) and expressed as micrograms per kilogram.

Multivariate analyses on the VOCs composition were performed using hierarchical clustering analysis (HCA) coupled with a heatmap on concentrations divided by the median and log_2_-transformed. The heatmap HCA was carried out with the R package gplots [41] using the Ward clustering method based on Euclidean distance.

### 2.7. Multiblock Data Integration

The fusion of six blocks of information, i.e., the data matrices related to the metabarcoding, sensory characteristics, VOCs, microbial enumeration, biogenic amines and TVBN, was undertaken. For each type of product, observations were available at 3 sampling dates (T0, T14 and T28) in 3 independent replicates. In the case of sensory analysis, only one observation was available (see Section 2.5) and duplicated. The microbial ecosystem through metabarcoding data was represented using the two dimensions of an MDS analysis (see Section 2.4). Among various approaches proposed for multiblock data analysis, the ComDim method was considered [42,43]. ComDim analysis stipulates that the various blocks share the same underlying common components but that these components may be differentially weighted. The specific weight, or salience, of each block on each common dimension was iteratively optimized during the algorithm. They highlighted the importance of the various blocks for the determination of the common dimensions.

## 3. Results

### 3.1. Microbial Analysis

**Figure 1:**
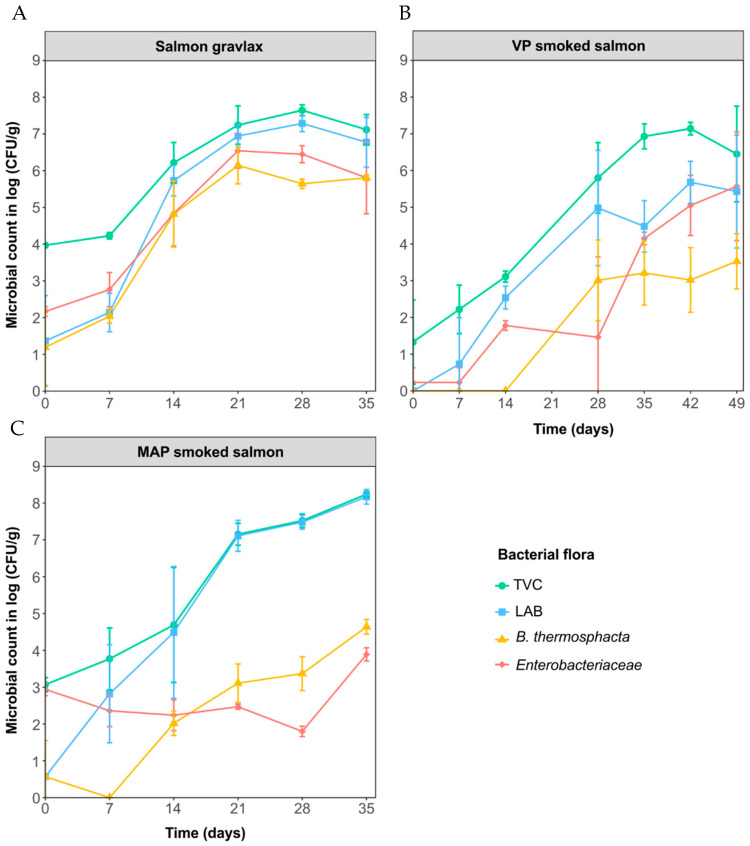
Microbial kinetics in the salmon gravlax (SG) (**A**), cold-smoked salmon packed under vacuum (VP CSS) (**B**) and cold-smoked salmon packed under modified atmosphere (MAP CSS) (**C**) during storage at 4 °C for 1 week and then at 8 °C. Bars represent the standard deviation of the mean of 3 replicates.

For the three products, the TVC, total LAB, Enterobacteriaceae and *B. thermosphacta* growth kinetics are shown in Figure 1. Globally, each enumerated flora started to increase quickly after 7 days, which corresponded to the temperature shift from 4 to 8 °C. The SG initial TVC count was higher than for MAP and VP CSS (4.0 ± 0.1, 3.1 ± 0.2 and <1.7 (detection threshold) log CFU/g, respectively. Maximum TVC counts were reached after 21 days for SG and 35 days for VP and MAP CSS (approximately 7, 7 and 8 CFU/g, respectively).

In the SG, the initial contamination could not be clearly defined as the TVC was 2–3 log CFU/g higher than the selective counts. LAB, Enterobacteriaceae and *B. thermosphacta,* although in a minority at T0, quickly grew and reached 6.9 ± 0.3, 6.5 ± 0.5 and 6.1 ± 0.5 log CFU/g, respectively, at T21. However, TVC still remained a little higher than LAB (+0.2 log CFU/g on average).

MAP CSS microbiota was dominated by LAB after 14 days of storage (LAB count equal to TVC and 3–4 log CFU/g higher than the other counts). The Enterobacteriaceae that were initially present (2.9 ± 0.2 log CFU/g) did not grow in the product. *B. thermosphacta*, which were not detected until 7 days, increased slowly to reach a maximum of 4.6 ± 0.2 log CFU/g after 35 days of storage.

The initial contamination of the VP CSS was lower than in the other products and remained so until 28 days. After 35 days, the maximum count was reached (6.9 ± 0.3 CFU/g) and was similar to the SG, but differed in composition. Indeed, LAB, Enterobacteriaceae and *B. thermosphacta* grew in the product but they never dominated. Their final concentrations were always 2 to 4 log CFU/g lower than the TVC (4.5 ± 0.7, 4.1 ± 0.2 and 3.2 ± 0.9 log CFU/g, respectively, after 35 days).

For all sampling dates, an important variability in microbial counts was observed independent of the microbial flora considered, in particular, in the case of the VP CSS. Fillets were cut lengthwise and slices were gradually packed, resulting in a plastic blister containing only slices from the head, belly or tail part of the fish. The heterogeneous thickness and fat content may therefore explain the variability within triplicates.

### 3.2. Ecosystem Monitoring through Metabarcoding

A total of 3,977,403 raw reads were obtained after the Illumina sequencing. A total of 1,746,144 reads passed through the FROGS pipeline for an average of 63,270 reads per sample. The number of sequences per sample ranged from 35,626 to 114,681 and the number of reads was normalized via rarefaction upon the lowest number of sequences per sample.

**Figure 2:**
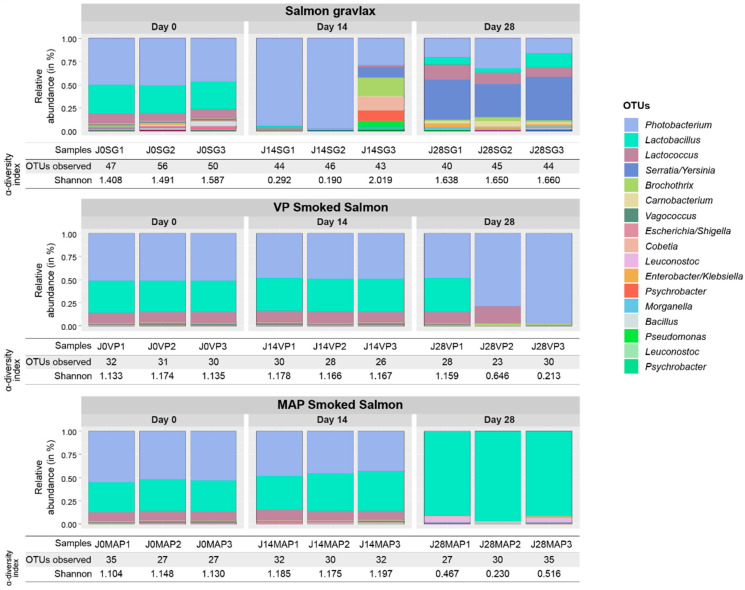
SG, MAP and VP CSS ecosystem relative compositions and α-diversities. Only OTU with a number of sequences representing more than 0.1% of the total number of reads appear in the legend and are ordered from the most abundant to the least abundant. The sample nomenclature is structured as follows: (1) sampling date, (2) type of sample (MAP, VP or SG) and (3) replicate number. For each sampling date, the experiments were done in triplicates.

The SG, MAP CSS and VP CSS ecosystem compositions over time are shown in Figure 2. Regardless of the product and the three sampling dates, the microbial diversity was quite low. The richness in terms of the number of OTU per sample ranged between 27 and 56. The evenness of the OTU abundance distribution, calculated with the Shannon index, was between 1.1 and 1.5 for almost all samples. The values that were calculated for these two indexes showed an ecosystem that was mainly composed of a small number of highly dominant OTU that were affiliated with nine main bacterial genera: *Photobacterium*, *Lactobacillus*, *Lactococcus*, *Serratia*/*Yersinia*, *Brochothrix*, *Carnobacterium*, *Vagococcus*, *Escherichia/Shigella* and *Cobetia*, with a representative number of sequences that was superior to 0.5% of the total reads.

**Figure 3:**
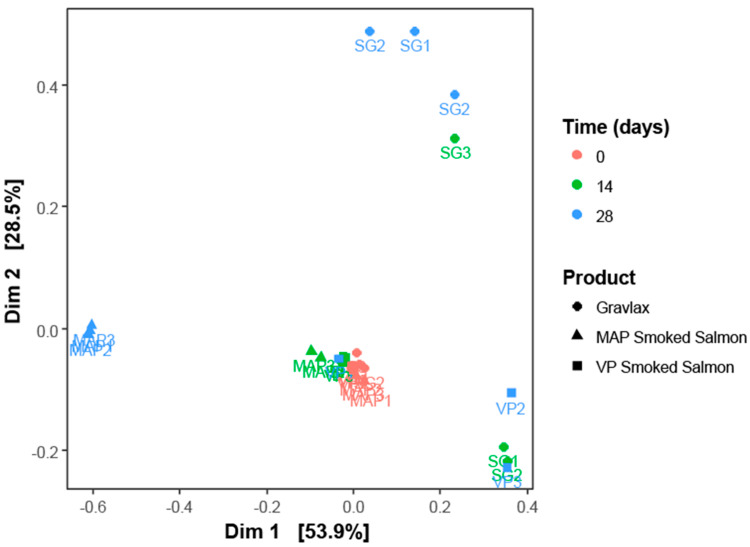
Multidimentional scaling representation of the SG, MAP and VP CSS samples microbial composition based on Bray–Curtis distances. The sample nomenclature is structured as follows: (1) the type of product (SG, MAP or VP) and (2) the replicate number.

At the beginning of storage, the ecosystems were highly similar between the products, as the samples were closely clustered together on the MDS ordination plot shown in Figure 3. *Photobacterium*, *Lactobacillus* and *Lactococcus* were the dominant genera, accounting for about 95% of the microbial composition, with 50, 29–34 and 8–11%, respectively (Figure 2). However, some differences were observed between the products. The SG initial microbial diversity was slightly higher than in the smoked products and remained higher until the end of storage. Indeed, twenty OTU that were specific to the gravlax were found, representing the fifteen following bacterial or archaea genera: *Halorubrum*, *Shewanella*, *Bacillus*, *Weissella*, *Hafnia*/*Obesumbacterium*, *Acinetobacter*, *Brachybacterium*, *Duganella*, *Terribacillus*, *Spelaeicoccus*, *Halohasta*, *Staphylococcus*, *Comamonas*, *Sphingobacterium* and *Brevibacterium*. *Staphylococcus* and *Acinetobacter* were the only shared genera between the three products (genera represented by several OTU). Genera such as *Halohasta*, *Staphylococcus* and *Comamomas*, which were specific to the SG microbial ecosystem as earlier reported were detected until 28 days of storage. Conversely, a differential abundance analysis using the DESeq2 R package revealed no statistical difference between the MAP and VP CSS microbial compositions at day 0.

After 14 days of storage, the gravlax microbial ecosystem was the only one that diverged from the first sampling date, with a diversity that decreased significantly over time (ANOVA on the richness index, *p*-value = 0.045). Except for the third biological replicate, the ecosystem composition was largely dominated by *Photobacterium*, whose relative abundance increased from 50 to 97.5% (Figure 2). In the third replicate, the microbial composition was more evenly distributed (highest Shannon index of 2.0) between seven genera: *Photobacterium* (29.0%), *Brochothrix* (19.6%), *Cobetia* (14.4%), *Psychrobacter* (13.8%), *Serratia/Yersinia* (11.9%), *Pseudomonas* (5.6%) and *Lactococcus* (1.7%). In the case of the MAP and VP CSS, no statistical differences in terms of the OTU abundance were noticed between days 0 and 14 (Figure 2 and Figure 3). Only a slight increase in abundance of 2 OTU belonging to *Leuconostoc* genera was observed for the MAP CSS. Moreover, in contrast to gravlax, the storage time showed no statistical effect on the ecosystem richness of the two smoked products (ANOVA, *p*-values of 0.854 and 0.196).

For the three products, the most important shift in the ecosystem took place between 14 and 28 days of storage (Figure 3). After 28 days, the SG ecosystem composition was more diverse than the two smoked products (Figure 2). The *Photobacterium* abundance dropped from 97.5 to 16.5–32.6% (variation between the triplicates) and Enterobacteriaceae increased to reach 35.1–46.9% for *Serratia*/*Yersinia* and 1.7–6.0% for *Enterobacter*/*Klebsiella*. Some LAB genera, such as *Lactobacillus*, *Carnobacterium* and *Lactococcus*, which were weakly present at 14 days (<1.5%), also increased after 28 days to reach 4.1–14.3, 1.9–6.6 and 10.8–16.4%, respectively. For the MAP CSS, *Photobacterium* and *Lactococcus* disappeared (<0.5% of abundance) in favor of *Lactobacillus*, which became the only dominant genus with more than 90% abundance and, to a lesser extent, *Leuconostoc* (1 to 5% abundance). Conversely, for VP CSS, despite the variability between replicates, the *Photobacterium* abundance increased to reach 78.4 and 96.8% in the second and third replicates, respectively, although *Lactococcus* and *Lactobacillus* remained present in some replicates.

### 3.3. Salmon Based Products Sensory Evolution

**Figure 4:**
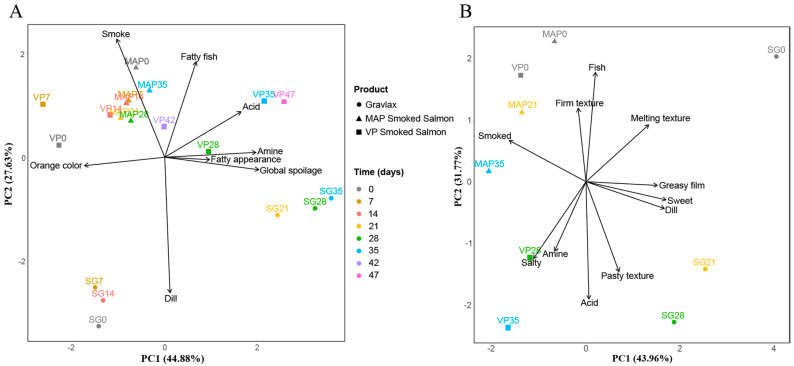
Normalized 2D principal component analysis (PCA) representation of the salmon gravlax (SG), smoked salmon stored under a modified atmosphere (MAP) and smoked salmon under vacuum package (VP) sensory evolutions based on visual and odor descriptors (**A**) and flavor and texture (**B**). Numbers associated with the sample name correspond to the sampling date in days.

Until the end of the experiment, none of the products exceeded the sensory rejection threshold (global spoilage score < 6). The spoilage was weak, with maximum scores of 3.9, 0.5 and 2.7 for the SG, MAP CSS and VP CSS, respectively, at the end of the experiment (data not shown). The PCA that was performed on the scores of profiling tests presented the main sensory characteristics for all samples (Figure 4). In Figure 4A (odor/color), the first axis (44.9% inertia) discriminated unspoiled from lightly spoiled samples, while the second (27.6% inertia) discriminated unsmoked (SG) from smoked samples (MAP and VP CSS). In Figure 4B (flavor and texture), it was the opposite, with axis 1 (43.9% inertia) discriminating SG from smoked products and axis 2 (31.7% inertia) discriminating unspoiled from lightly spoiled samples.

The SG was considered lightly spoiled after 21 days, with the global spoilage score increasing from 0.2 on day 0, to 3.0 and 3.9 on days 21 and 35, respectively (data not shown). The gravlax spoilage was defined by a significant decrease in the perception of the dill odor, orange color, fish flavor and melting texture (Figure 4). Salty flavor and amine and acid flavor and odor significantly increased (Duncan test with *p*-value < 0.05). However, the acidic and amine odors that were associated with spoilage never exceeded scores of 2.0 and 2.6, respectively, after 35 days of storage.

The MAP CSS was never considered spoiled. During the 35 days of storage, it was characterized by a high perception of smoke odor and flavor, a stable fatty fish odor and flavor, a firm texture with moderate development of fatty droplets (greasy film) and the absence of spoilage notes, such as amine odor and flavor (score < 0.5) (Figure 4). The MAP CSS nevertheless showed some minor sensory changes during storage. Smoke odor, orange color and melting texture significantly decreased after the first week of storage, while the salty and acid flavor lightly increased after 35 days (*p*-value < 0.05). A strong butter odor that was not figured in the sensory analysis criteria was also detected by panelists at the first sampling date and was never found later. 

The VP CSS was considered lightly spoiled after 35 days of storage with a score of 2.5. The smoke flavor and odor intensity were highly perceived by panelists at the beginning of storage and decreased at the end. As for the MAP CSS, the panelists also described the presence of a strong butter odor, which disappeared after the first week. The orange color and the firmness of the product also decreased significantly after 28 days of storage. An amine odor and flavor and an acidic flavor started to increase after 28 days to reach 2.8, 1.9 and 3.9, respectively. The fatty aspect of the product also increased significantly after 14 days (from 2.9 to 5.2 after 49 days). Nevertheless, no significant difference during storage was found for the greasy film texture (*p*-value = 0.31). An increase in the pasty texture, although not significant (*p*-value = 0.08), was also observed after 28 days of storage.

### 3.4. Chemical Analyses

The pHs of the three products were almost identical and remained stable during the whole storage time, with values between 5.47 and 5.93 (Table 1). However, the SG initial pH (5.87 ± 0.10) was slightly higher (*p*-value < 0.05) than the MAP and VP CSS initial pHs (5.68 ± 0.02 and 5.63 ± 0.03, respectively). The initial TVBN contents were similar for the three products (12.0 ± 1.8 to 14.5 ± 1.6 mg-N/100 g (Table 1). The TVBN concentration in the SG and VP CSS increased during storage and reached their maxima of 28.1 ± 1.0 mg-N/100 g at day 28 and 24.7 ± 2.5 mg-N/100 g at day 35, respectively. No production of TVBN in the MAP CSS was observed until the end of the experiment. Regardless of the product, no TMA was detected during the storage.

### 3.5. Biogenic Amines Content

Tryptamine, 2-phenylethylamine, putrescine, spermidine and spermine were under the detection threshold in all samples. The concentrations of cadaverine, tyramine and histamine are presented in Table 1. High variability was observed within the triplicates.

Concentrations of the three biogenic amines in the SG started to increase between 7 and 14 days of storage and remained stable until 28 days within a range of 276 ± 31 to 404 ± 79 mg/kg for cadaverine, 42 ± 13 to 65 ± 61 mg/kg for tyramine and 56 ± 7 to 117 ± 104 mg/kg for histamine. As for the TVBN content, a decrease in biogenic amines was observed after 35 days.

In the VP CSS, biogenic amines production started after 28 days and also remained more or less stable until day 49. Their respective range values were always lower than in the SG and ranged from 153 ± 160 to 219 ± 188 mg/kg for cadaverine, 27 ± 36 to 49 ± 78 mg/kg for tyramine and 11 ± 6 to 59 ± 45 mg/kg for histamine. Although the mean concentration of histamine was lower than 100 mg/kg (European tolerated concentration in some fish species), some replicates exceeded this limit (SG at day 14, VP CSS at days 35 and 49; data not shown).

In the MAP CSS, no biogenic amine exceeded 10 mg/kg for the whole storage duration.

### 3.6. VOCs Profile

**Figure 5:**
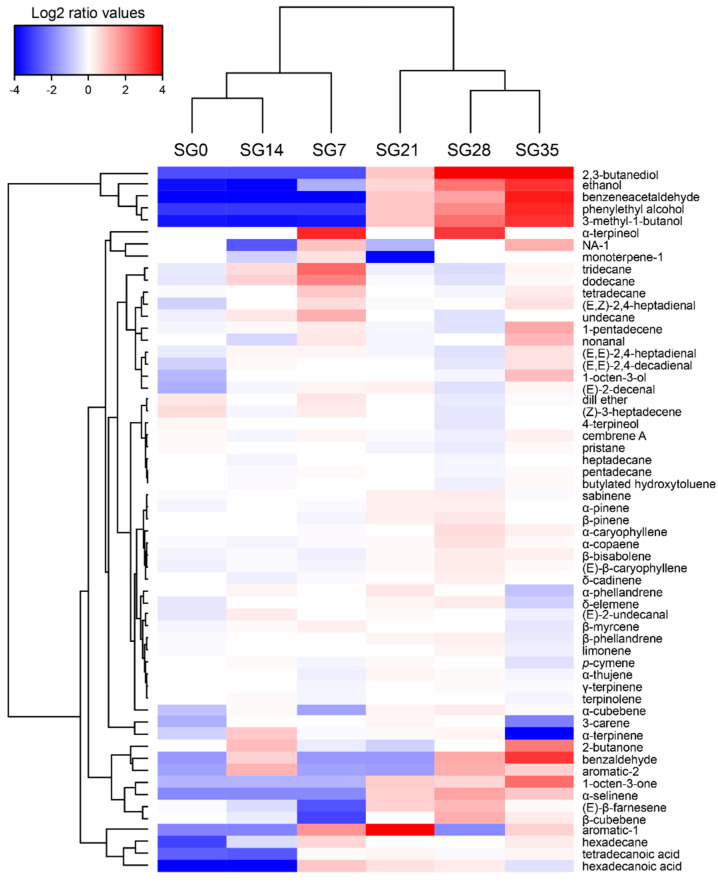
SG VOC hierarchical cluster analysis (HCA) heatmap based on the Euclidean distance calculated from log_2_(n) transformation of the mean concentration (n = 3) amended by the median. The sampling time points are represented in columns (0 to 35 days), while the VOCs are depicted in rows. Blue colors indicate lower metabolite concentrations, while red colors show higher metabolite levels. See Supplementary Table S1.

**Figure 6:**
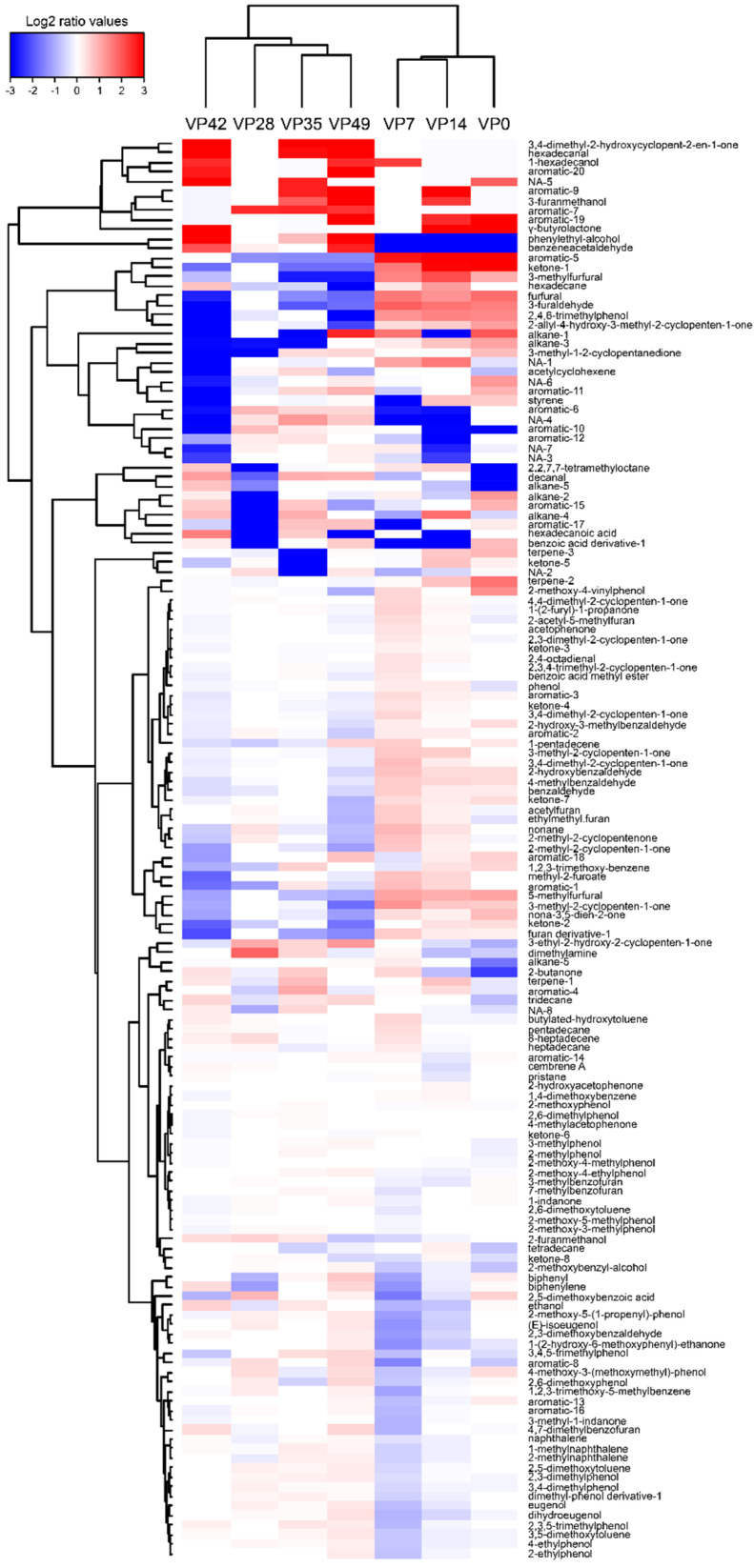
VP CSS VOC hierarchical cluster analysis (HCA) heatmap based on the Euclidean distance calculated from a log_2_(n) transformation of the mean concentration (n = 3) amended by the median. The sampling time points are represented in columns (0 to 35 days), while the VOCs are depicted in rows. Blue colors indicate lower metabolite concentrations, while red colors show higher metabolite levels. See Supplementary Table S1.

**Figure 7:**
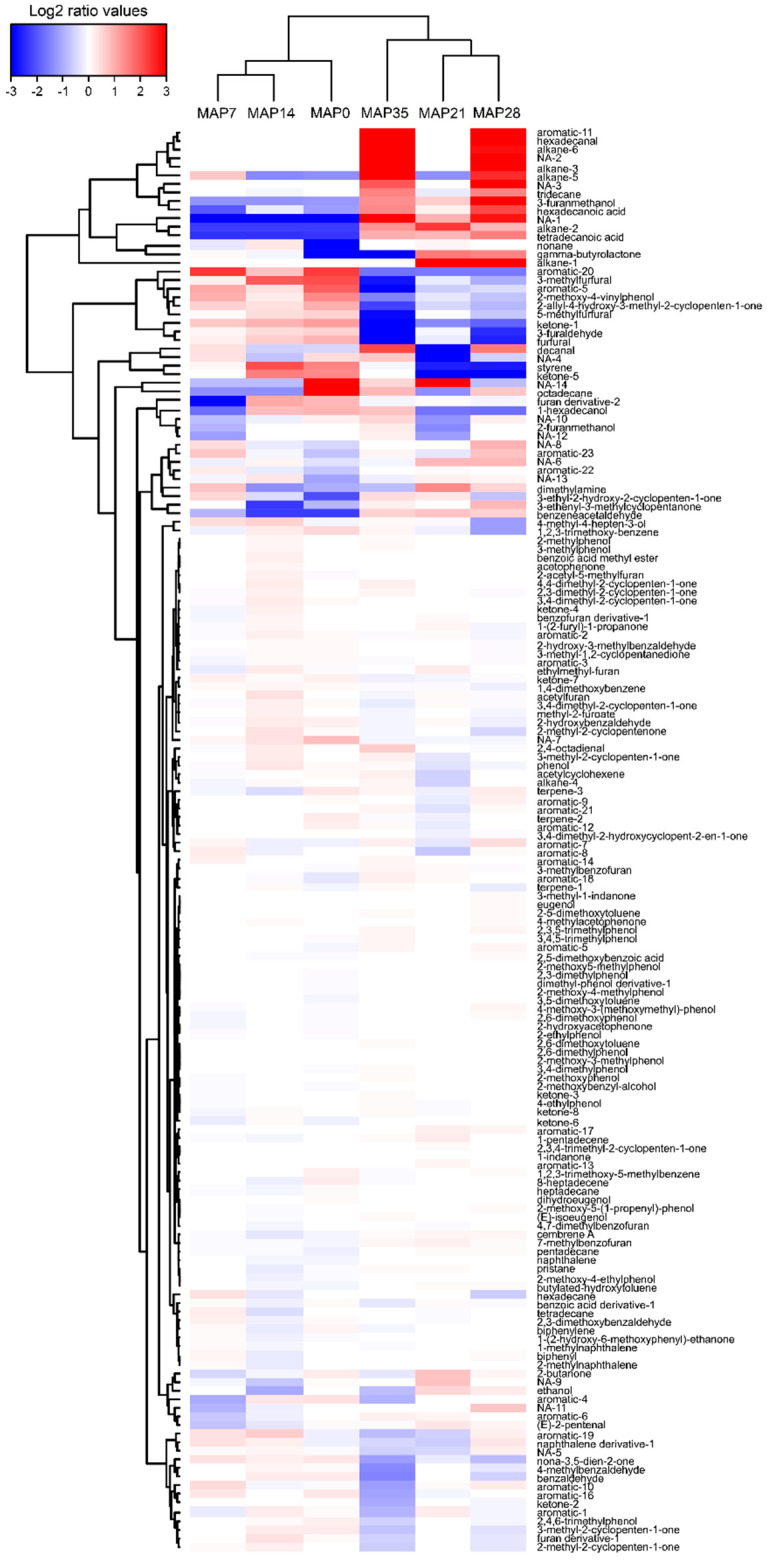
MAP CSS VOC hierarchical cluster analysis (HCA) heatmap based on the Euclidean distance calculated from a log_2_(n) transformation of the mean concentration (n = 3) amended by the median. The sampling time points are represented in columns (0 to 35 days), while the VOCs are depicted in rows. Blue colors indicate lower metabolite concentrations, while red colors show higher metabolite levels. See Supplementary Table S1.

The evolution of the volatilome composition for each product was visualized with an HCA heatmap based on a log_2_(n) ratio of the mean concentrations amended by the median value (Figure 5, Figure 6 and Figure 7).

The SG volatilome composition showed the lowest complexity, with 59 compounds detected using SPME-GC/MS. Twenty-eight were identified as terpenes, which were probably related to the presence of the spices (black pepper, dill) used in the curing step, where their concentrations remained stable during the storage period. As the storage progressed, an increase in the concentrations of alcohols (ethanol, 3-methyl-1-butanol, 2,3-butanediol, 1-octen-3-ol, phenylethyl alcohol), aldehydes (nonanal, (E,E)-2,4-decadienal), aromatic aldehydes (benzaldehyde, benzeneacetaldehyde), ketones (2-butanone, 1-octen-3-one) and 1-pentadecene was visible (Figure 5). Conversely, the alkane concentrations of, e.g., undecane, dodecane, tridecane and tetradecane, decreased over time.

The MAP and VP CSS volatilome compositions were much more complex than in the gravlax, with 164 and 149 compounds detected, respectively. For both products, more than half of all compounds were identified as furan and aromatic compounds that were related to the smoking process.

In the case of the VP CSS, despite the heterogeneity between the samples, a trend emerged that was characterized by an increase in the concentration of some aldehydes (decanal, hexadecanal), aromatic aldehydes (benzaldehyde, benzeneacetaldehyde), phenylethyl alcohol and 3,4-dimethyl-2-hydroxycyclopent-2-en-1-one (Figure 6). An increase in the production of dimethylamine (at days 28 and 35) and ethanol (at days 35 and 42) was also visible. A decrease in the concentrations of some furan and aromatic compounds was recorded, such as 2,4,6-trimethylphenol, furfural, acetylfuran, 3-methylfurfural, 5-methylfurfural and unidentified aromatic-5/19/20.

Despite the absence of spoilage, slight variations in the MAP CSS VOCs concentrations were observed during storage. An increase in tetradecanoic and hexadecanoic acid, ethanol (at days 21 and 28), decanal, hexadecanal (after 28 days), tridecane, unidentified alkane-1/2/3/5/6, 3-furanmethanol and dimethylamine (at days 7, 21 and 28) were found (Figure 7). Conversely, a decrease in the concentration over time was visible for 3-furaldehyde, styrene, unidentified aromatic-5/11/20 and 2-methoxy-4-vinylphenol. As for the VP CSS, a decrease in the concentrations of furfural, 3-methylfurfural and 5-methylfurfural was also found. Nonetheless, except for furan and the aromatic compounds, the concentrations were relatively low (Supplementary Table S2); consequently, the variations that were observed in the HCA heatmap representation were amplified by the median emendation and the ratio transformation in log_2_(n).

### 3.7. Integration of the Various Blocks of Data

ComDim was performed using the six blocks of data: the microbial ecosystem data (metabarcoding) through the two MDS dimensions of the OTU abundance analysis (“OTU.dim”, two variables), the sensory data (“SENSO”, eight variables of odor/color), the log_2_(n)-transformed VOC data (“log2VOC”, 17 compounds in common), the microbiological data (“MICROBIO”, six variables), the biogenic amines (“BIOGAMI”, six variables) and the TVBN measurements (one variable). 

From Table 2, three dimensions were retained. The first dimension accounted for 45% of the total variation in the multiblock data set. For this dimension, TVBN had the highest specific weight, or salience (52%), followed by the blocks BIOGAMI (22%) and MICROBIO (18%) (data not shown). The second dimension was specifically associated with the block OTUdim (salience of 91%), whereas the third dimension accounted for both the log_2_VOC and SENSO blocks (saliences of 52 and 41%, respectively). 

**Figure 8:**
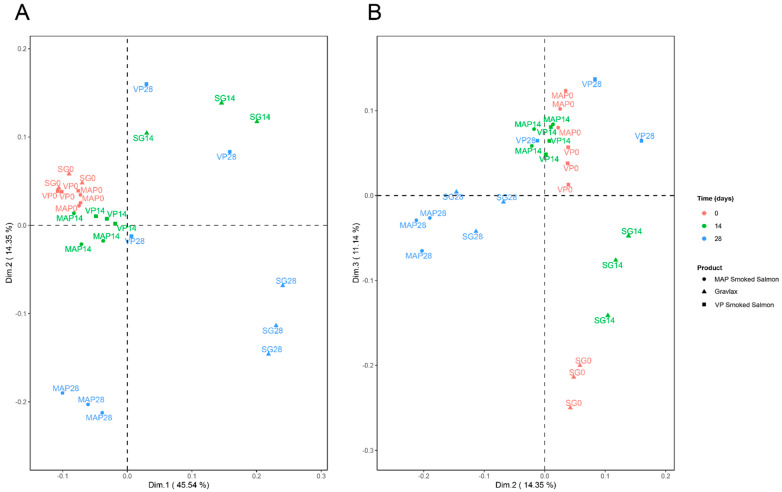
Multidimensional representation of the three products, namely, SG, MAP CSS and VP CSS, at three sampling time points using the various blocks of data collected by means of the ComDim method. (**A**) Projection of individuals in dimensions 1 and 2, (**B**) projection of individuals in dimensions 2 and 3. Numbers associated with the sample name correspond to the sampling date in days.

The score plots in dimensions 1 and 2, on the one hand, and 2 and 3, on the other hand, are shown in Figure 8A,B. The evolution of the products with the number of days of storage was reflected in different ways. For the SG at 28 days and, to a lesser extent, for the SG at 14 days or VP at 28 days, the increase in the TVBN (along the first dimension) was pronounced. By comparison, the MAP CSS did not show such a pattern at 28 days, but a modification in the microbial ecosystems was noticed, which was reflected by the second dimension. As stated in Section 3.2, the MAP ecosystem composition was characterized by a decrease in *Photobacterium* in favor of *Lactobacillus* over time, which was expressed along the second dimension of ComDim. Moreover, a detailed analysis of the OTU with this second dimension made it possible to highlight that the SG ecosystem at 28 days of storage could be associated with specific genera, such as *Carnobacterium*, *Morganella* and *Bacillus*. Finally, the third dimension of ComDim showed a common contribution of sensory and VOCs data, allowing for the description of SG salmon samples at 0 or 14 days of storage, which was not shown for the MAP and VP CCS. The correlation coefficients between the raw data and this third dimension highlighted the sensory odor “dill” as being specific toward SG at day 0, which was associated with VOCs, such as pristane, pentadecane, heptadecane and tridecane. A smoke odor was associated with benzaldehyde and butylate hydroxytoluene compounds.

## 4. Discussion

The objective of this study was to gain deeper knowledge about the quality of three salmon-based products and to compare the effect of the manufacturing process on their microbial ecosystem, organoleptic properties and volatilome. After the packaging step, the TVC counts were 4.0, 3.0 and <1.7 log CFU/g for the SG, MAP CSS and VP CSS, respectively. Such initial bacterial loads are usually found in lightly preserved salmon products [3,4,5,6,9,14,44] but may sometimes reach 5–6 log CFU/g depending on the raw material and smokehouse hygienic quality [4,6,10]. The difference within the product was more likely due to the smoking process rather than the salt concentration, which was lower in the less-contaminated product (2.58 ± 0.05, 3.05 ± 0.12 and 2.17 ± 0.28 NaCl/100 g flesh for the SG, MAP CSS and VP CSS, respectively). Phenolic compounds are known to possess antimicrobial activity [45] and are responsible for bacterial growth delay or inhibition in CSS [46,47,48,49]. In this study, the total phenolic compounds were much more concentrated in the MAP and VP CSS (28 ± 1 and 35 ± 5 mg/kg of flesh, respectively) than what is usually found in CSS (often < 10 mg/kg). The lower phenolic concentration in MAP CSS compared to VP CSS may explain the higher initial bacterial count in the MAP CSS. This may also be explained by the supplementary cutting step to obtain dices, which was probably responsible for the recontamination before packaging, especially by Enterobacteriaceae (Figure 1).

On day 0, the three products’ microbial ecosystems were relatively close in the proportions of *Photobacterium*, *Lactobacillus* and *Lactococcus*, accounting for about 50, 30 and 10% of the composition, respectively. Despite an overall compositional similarity, the SG ecosystem diversity was initially higher than the smoked products and remained so until 28 days. The diversity may be explained by the spices and herbs used for the SG preparation and the absence of selective pressure due to smoke. There were 20 OTU specific to the SG and belonged to the bacterial and archaeal kingdom. Among them, *Shewanella (S. putrefaciens*, *S. vesiculosa*, *S. morhuae*, *S. baltica*, *S. frigidimarina)*, *Hafnia (H. alvei)*, *Weissella* (*W. confusa*, *W. viridescens*, *W. kandleri*), *Staphylococcus* (*S. equorum*) and *Bacillus* are ubiquitous bacterial genera that were isolated from a wide variety of ecological niches, including seafood [3,6,9,13,14,17,18,19,50,51,52,53,54,55,56,57,58]. On the other hand, for some ecological niches, *Duganella*, *Terribacillus*, *Spelaeicoccus*, *Comamonas*, *Sphingobacterium*, *Brevibacterium*, *Halorubrum* and *Halohasta* are relatively unknown and not frequently described. Table 3 summarizes some ecological origins of these uncommon bacteria or archaea. Some were isolated from a specific ecological niche (e.g., *Halohasta* and *Halorubrum* in salt) but others are ubiquitous and can therefore be brought with the spices used for the SG preparation. Next-generation sequencing techniques are powerful tools for pinpointing minor microbial populations within ecosystems, which is information that is otherwise inaccessible using common cultural methods. For instance, Chaillou et al. [17] also detected *Sphingobacterium* sp. and *Brachybacterium* sp. in diced bacon and *Comamonas* sp. in cod and salmon fillets with pyrosequencing technology, although no isolate was collected.

For the three matrices, the most important shift in the microbial population occurred between 14 and 28 days, when the growth of different bacterial flora reached their stationary phase. After 28 days, in the SG, Enterobacteriaceae mostly dominated the ecosystem, with an OTU corresponding to the *Serratia/Yersinia* genera representing 35–47% of the total composition. In trout gravlax stored under VP at 3 and 8 °C, Lyhs et al. [15] showed that H_2_S-producing bacteria (which include some Enterobacteriaceae) represented a major part of the spoilage microbial flora. Wiernasz et al. [16] also found *Serratia/Yersinia* in SG on days 14 and 21 with metabarcoding analysis. Smoke seemed to exert an additional selection pressure during storage as the OTU number was lower in the VP CSS and MAP CSS than in the SG. At the end of the storage, the microbial composition of the MAP CSS was only dominated by LAB, such as *Lactobacillus* (>90%) and, to a lesser extent, *Leuconostoc* (4–6%). These results were in accordance with those obtained with the cultural method, with the LAB count being identical to the TVC. In the VP CSS, depending on the biological replicate, *Lactobacillus* and *Lactococcus* were also detected in a high proportion (13 to 37%). The VP and MAP of food commodities are known to favor LAB growth [13,59,60,61]. *Lactobacillus* and *Lactococcus* were the main LAB genera that were detected in the products. Many species of *Lactobacillus* (*L. alimentarium*, *L. casei* subsp. *tolerans*, *L. coryneformis*, *L. curvatus*, *L. delbrueckii* subsp. *delbrueckii*, *L. farciminis*, *L. fuchuensis*, *L. homohiochii*, *L. malfermentans*, *L. plantarum*, *L. pentosus*, *L. brevis*, *L. sakei* and *L. sanfranciscensis*) and *Lactococcus* (mainly *L. piscium*, but also *L. lactis* and *L plantarum*) are frequently isolated from seafood products [17,62,63,64]. Although the spoilage potential of LAB in seafood is strain-dependent, some species, such *L. sakei*, *L. farciminis* and *L. fuchuensis*, frequently induced off-odor in shrimp, raw salmon and CSS. Other species (*L. alimentarius*, *L. piscium*) generally do not affect the quality [18,63,65,66,67]. No sign of alteration was found in the MAP CSS, even after 35 days. This may have been due to the absence of such spoilage species. However, the relatively low concentration of LAB (10^7^ CFU/g) may also explain this result.

In the VP CSS, the *Photobacterium* relative abundance increased from day 14 to day 28 and was dominant, accounting for 78 to 97% of the microbial composition. *Photobacterium* is capable of anaerobic respiration via the use of TMA-oxide, which is often present in marine fish, as a final electron acceptor, making this bacterium able to grow under MAP and VP [50]. The final metabolite, namely, the TMA, is strongly malodorant and responsible for “amine” off-odors. *Photobacterium* was also found to be part of the dominant microbial flora of VP CSS [12,18]. However, caution must be taken with a metabarcoding approach as its detection might be largely overestimated due to the use of the 16S rRNA gene as a target. According to the rrnDB database [68], *Photobacterium*, especially *P. phosphoreum*, which is the main species encountered in seafood, possesses 15 copies of the 16S rRNA gene in its genome, while the average copy number for the bacterial kingdom is only 4.8. 

The three products’ sensory qualities remained acceptable for the whole storage duration. Usually, lightly preserved seafood products are declared spoiled and unfit for consumption after 3 to 4 weeks for fish gravlax [14,15] and after 5 to 6 weeks for CSS [3,4,5,6,9,10], and their final bacterial load can reach 8 to 9 log CFU/g [13]. In our study, the extended shelf-life might be explained by the low TVC count, which never exceeded 7–8 log CFU/g, which was probably related to high salt and smoke concentrations. Weak spoilage, as well as acidic and amine off-odors and flavors, increased a bit during storage of the SG and VP CSS, as well as fatty droplets at the products’ surfaces, while the perception of the freshness-related odor, flavor and color, such as fresh fish, dill, smoke and orange, decreased slightly over time. Such spoilage sensory characteristics are commonly described for lightly preserved salmon products [5,6,10,69]. They can be induced by the presence of specific spoiling bacteria, such as *B. thermosphacta*, *P. phosphoreum* and *S. liquefaciens* [65]. The fact that spoilage was classified in the following order: SG > VP CSS > MAP CSS might be explained by the microbial diversity and higher presence of Gram-negative bacteria, such as *P. phosphoreum* and Enterobacteriaceae.

Resulting from enzymatic reactions and microbial degradation, the TVBN [4,10,70] and biogenic amines [51,71,72] can be indicators of spoilage in seafood. In the SG and VP CSS, the TVBN, tyramine, cadaverine and histamine production started when the TVC count reached 6 log CFU/g after 14 and 28 days for the SG and VP CSS, respectively. Although not known to be a fish with a high histidine content, some replicates of each product exceeded 100 mg/kg of histamine, which is the EU regulatory tolerated concentration in some fish species (not including salmon) [73]. This may have been related to the presence of *Photobacterium* sp. Some authors have found equivalent or even higher concentrations for cadaverine, tyramine and histamine in spoiled CSS [51,69]. No production of biogenic amines was found in MAP CSS, despite a microbial load of 8.2 ± 0.1 log CFU/g. In this product, the microbial ecosystem was mainly dominated by *Lactobacillus*, which are not strong biogenic amine producers in seafood [74,75]. In the same way, no TVBN production was found in the MAP CSS. In the SG and VP CSS, the TVBN increased slightly over time but did not exceed the European regulatory limit of 35 mg-N/100g for unprocessed salmon [73].

The VOCs composition of the three products, especially the MAP CSS, did not change much over time. However, increases in the concentrations of alcohols (ethanol, 3-methyl-1-butanol, 2,3-butanediol, 1-octen-3-ol, phenylethyl alcohol), aldehydes (nonanal, (E,E)-2,4-decadienal, hexadecanal benzaldehyde, benzeneacetaldehyde), ketones (2-butanone, 1-octen-3-one) and dimethylamine (VP CSS only) were observed. Aldehydes, amines, ketones, alcohols and organic acids production result mostly from microbial activity and their concentration increased concomitantly with the deterioration of the seafood organoleptic properties [8,76,77,78,79]. For instance, aldehydes resulting from lipids oxidation by microorganisms are good indicators for food degradation and actively participate in the characteristic rancid, cooked potatoes, fatty, floral, fruity and grassy odors of spoiled fish [8,78,79]. *P. phosporeum*, *B. thermosphacta* and *S. liquefaciens*, which are common seafood spoilers, induced the production of many spoilage-related volatile compounds, such as benzene ethanol, 2-methylpropanol, 3-methyl-2-butanol, 2,3-methyl-1-butanol, 1-propanol, 1-penten-3-ol, acetaldehyde, 2,3-methyl-1-butanal, pentanal, 2-methyl-1-propanal, benzaldehyde, benzeneacetaldehyde, 2,3-butanedione, 2-propanone, 3-pentanone, 3-methyl-2-butanone, 3-hydroxybutanone (acetoin), acetic acid and ethyl acetate, when added in cooked and peeled shrimp and raw and cold-smoked salmon [36,80,81]. Interestingly, an important decrease in furanic aldehydes (furfural, 3-methylfurfural, 5-methylfurfural and 3-furaldehyde) was observed during the storage of the two smoked products. Many furanic aldehydes are found in smoked fish [8,77,79,82,83]. Most of them are found in the smoke but can also be generated through Maillard and Strecker reactions between the wood smoke and the fish flesh during the smoking process [78]. Several bacteria, including species from *Pseudomonas*, *Acinetobacter* and *Serratia* genera, are able to metabolize furfural and hydroxymethylfurfural compounds into furfuryl alcohols (2-furanmethanol and 3-furanmethanol) [84]. These two alcohols were detected in the MAP and VP CSS and slightly increased over time.

**Table 3:** Ecological origin of uncommon genera that were only detected using the metabarcoding approach in salmon dill gravlax.

Genus	Ecological Origin	References
**Bacteria**		
*Brachybacterium*	Salt-fermented seafood, lake sediment, soil, seawater, animal feces	[85,86,87,88]
*Duganella*	Soil, plant roots, water	[89,90,91]
*Terribacillus*	Soil, salted lake sediment, plant material	[92,93,94]
*Spelaicoccus*	Soil	[95]
*Brevibacterium*	Soil, seawater, feces, compost, plant material, milk, cheese, poultry, CSS	[96,97,98,99,100]
*Comamonas*	Freshwater, plant, compost, soil, fish gut	[101,102,103,104,105]
*Sphingobacterium*	Soil, permafrost, glacier, animal feces and gut, milk, compost, plant material, lichen, freshwater	[106,107,108,109,110,111,112]
**Archaea**		
*Halorubrum*	Salt-fermented seafood, rock salt, salted lake sediment, solar saltern	[113,114,115,116]
*Halohasta*	Salted lake water, aquaculture water	[117]

The characterization of the product quality over the storage time involves the combination of a wide range of analyses giving heterogeneous sets of data requiring multivariate statistical analysis. In this study, the use of the multiblock analysis method ComDim allowed for characterizing the samples according to relationships existing between classical microbial analysis, microbiota, physico-chemical parameters, sensory analysis and VOCs. The main parameters that allowed for discriminating between samples were the TVBN and, to a lesser extent, the biogenic amines and microbial ecosystem. The SG on days 14 and 28 and the VP CSS on day 28 were mainly characterized by higher TVBN and biogenic amines contents. The MAP CSS on day 28 differed from the other samples mainly by its microbiological shift, with a decrease in *Photobacterium* and an increase in *Lactobacillus*, while some OTU were very specific to the SG. Sensory analysis and COV mainly discriminated samples at the early stage of storage according to the process (unsmoked from smoked products). The ComDim approach was used to illustrate the tools that could be used to integrate very heterogeneous data, especially those from metabarcoding, which is a relatively new technique. Such methods might be applied to larger sets of data that are derived from a larger number of samples showing less processing variability.

## 5. Conclusions

This study showed that the process and packaging conditions both had an effect on the microbial composition and the quality of the final product. Although the sensory spoilage was generally weak, gravlax was the most perishable product, followed by the VP CSS and MAP CSS. This result is in accordance with all the parameters measured in this study. The TVBN, cadaverine, tyramine and histamine were in higher concentrations in the SG, followed by the VP CSS and then the MAP CSS. The microbial diversity in gravlax was high, probably due to the spices and herb additions in the process and the absence of antimicrobial phenolic compounds. LAB aside, the most abundant genera that were identified using metabarcoding (*Photobacterium*, *Serratia/Yersinia* and *Brochothrix*) are known to be specific spoilage microorganisms in seafood. In the smoked product, after 28 days, the VP selected *Photobacterium* as the dominant flora but LAB, Enterobacteriaceae and *Brochothrix* were still present. The MAP selected only LAB (mainly *Lactobacillus*) as the dominant flora, which did not deteriorate the sensory quality.

## Figures and Tables

**Table 1:** pH evolution, TVBN (mg-N/100g) and biogenic amines (mg/kg) contents in the salmon gravlax (SG), vacuum-packed (VP) and modified atmosphere packed (MAP) CSS during storage at 4 °C for 1 week and then at 8 °C.

Sampling Date (days)	0	7	14	21	28	35	42	49
**SG**								
pH	5.87 ± 0.10	5.93 ± 0.02	5.62 ± 0.04	5.81 ± 0.03	5.77 ± 0.02	5.84 ± 0.02	-	-
TVBN	12.0 ± 1.8	15.1 ± 0.9	22.2 ± 5.3	24.9 ± 4.9	28.2 ± 1.0	23.1 ± 1.3	-	-
Cadaverine	2.0 ± 0.1	6.0 ± 3.6	294.2 ± 224.1	275.8 ± 30.6	404.1 ± 79.4	73.5 ± 58.2	-	-
Tyramine	2.0 ± 0.1	2.0 ± 0.1	65.3 ± 60.9	42.2 ± 12.9	63.5 ± 15.3	13.0 ± 12.0	-	-
Histamine	0.7 ± 0.6	1.3 ± 0.6	116.8 ± 104.3	57.7 ± 7	77.3 ± 24.0	17.1 ± 12.7	-	-
**MAP CSS**								
pH	5.68 ± 0.02	5.81 ± 0.01	5.53 ± 0.07	5.70 ± 0.02	5.65 ± 0.04	5.63 ± 0.02	-	-
TVBN	14.4 ± 0.7	14.6 ± 1.0	14.2 ±1.9	14.8 ± 1.0	13.3 ± 2.6	16.8 ± 2.1	-	-
Cadaverine	2.3 ± 2.1	4.7 ± 0.6	9.0 ± 5.6	6.7 ± 2.0	6.2 ± 1.0	4.4 ± 0.4	-	-
Tyramine	0.0	0.0	3.3 ± 3.1	0.2 ± 0.4	0.3 ± 0.5	6.2 ± 2.7	-	-
Histamine	3.7 ± 3.2	6.0 ± 1.0	8.0 ± 2.0	5.3 ± 0.6	5.5 ± 1.5	5.2 ± 0.7	-	-
**VP CSS**								
pH	5.63 ± 0.03	5.81 ± 0.02	5.47 ± 0.04	-	5.64 ± 0.07	5.76 ± 0.01	5.60 ± 0.05	5.57 ± 0.013
TVBN	14.5 ± 1.6	17.1 ± 1.3	17.5 ± 1.3	-	21.0 ± 3.7	24.7 ± 2.5	24.6 ± 3.6	25.9 ± 5.0
Cadaverine	4.0 ± 1.0	4.0 ± 0.1	4.6 ± 0.9	-	153.4 ± 160.5	185.0 ± 186.3	185.3 ± 281.7	218.9 ± 187.9
Tyramine	0.0	0.0	0.0	-	26.7 ± 36.0	37.8 ± 43.9	49.5 ± 78.5	38.8 ± 33.7
Histamine	6.7 ± 1.2	5.7 ± 0.6	5.6 ± 0.2	-	33.4 ± 29.6	56.3 ± 61.9	10.8 ± 6.0	59.4 ± 45.2

-: not determined.

**Table 2:** Percentage of the variability explained (%expl) and cumulative percentage (cum%expl) explained by the first common components defined using ComDim.

	%expl	cum%expl
Dim.1	45.56	45.56
Dim.2	14.35	59.91
Dim.3	11.15	71.06
Dim.4	6.45	77.51
Dim.5	4.15	81.66

## Supplementary Materials

The following are available online at https://www.mdpi.com/article/10.3390/foods10112517/s1. Table S1: OTU Table. Table S2: Volatile Organic Compounds Table.
